# Single-Pill Combination to Improve Hypertension Treatment: Pharmaceutical Industry Development

**DOI:** 10.3390/ijerph19074156

**Published:** 2022-03-31

**Authors:** Magdalena Paczkowska-Walendowska, Szymon Sip, Rafał Staszewski, Judyta Cielecka-Piontek

**Affiliations:** 1 Department of Pharmacognosy, Poznan University of Medical Sciences, Rokietnicka 3, 60-806 Poznan, Poland; mpaczkowska@ump.edu.pl (M.P.-W.); szymonsip@ump.edu.pl (S.S.); 2 Department of Hypertension, Angiology and Internal Medicine, Poznan University of Medical Sciences, Długa 1/2, 61-848 Poznań, Poland; rafal.staszewski@ump.edu.pl

**Keywords:** hypertension, fixed-dose combination, beta-blockers, ACE inhibitors, single-pill combination

## Abstract

Multiple illness is an increasingly common phenomenon. Its consequence is the need for polytherapy, which is particularly common among people suffering from arterial hypertension. The development of combined preparations (containing at least two API-active pharmaceutical ingredients) dedicated to the treatment of hypertension is a response to increased compliance, especially in elderly patients. In our work, we describe in particular the possibilities of using β-adrenergic receptors blockers and angiotensin-converting enzyme inhibitors in combinations. The combinations of APIs are used as single pills in patients with arterial hypertension with concomitant diseases such as hyperlipidemia; blood coagulation problems and diabetes mellitus were also discussed successively. Pharmacoeconomic analysis for the API combinations shown is also presented. As a final conclusion, numerous benefits of using the combined preparations should be indicated, especially by the elderly and/or in patients with coexistence of other diseases.

## 1. Introduction

Arterial hypertension remains the most important modifiable risk factor for cardiovascular disease, and according to the World Health Organization (WHO), it is still the world’s leading cause of premature death. According to the ESC/ESH Guidelines, hypertension is defined as a systolic blood pressure (SBP) of at least 140 mmHg and/or diastolic blood pressure (DBP) of at least 90 mmHg, with grades 1–3 hypertension [1].

The blood pressure level (BP) shows a linear relationship with mortality and incidence of cardiovascular diseases (e.g., heart attack, stroke, or peripheral arterial disease) and renal failure, in all age and ethnic groups, both in women and men. In 2015, it was predicted that 1.13 billion people worldwide have hypertension, with over 150 million in Central and Eastern Europe. According to WHO data, one in four men (25%) and one in five women (20%) had high blood pressure in 2015 [2]. With age, arterial hypertension becomes more frequent and occurs in over 60% of people over 60. As the population ages, adopts a more sedentary lifestyle, and increases weight, the incidence of high blood pressure will continue to increase worldwide. By 2025, it is expected that the number of persons with hypertension would have increased by 15–20%, to over 1.5 billion [3]. Economically, hypertension costs the US nation about $51.2 billion annually, and the total number of cardiovascular diseases cost the country about $316.1 billion annually in 2012–2013 [4]. As a result, lowering the health and economic costs of hypertension and cardiovascular disease is a top objective for public health.

In addition to the apparent side effects caused by hypertension and other heart diseases, the additional effects must result from polypharmacy. The increase in the hypertension prevalence with the patient’s age may increase the polypharmacy risk because elderly patients most often suffer from more than one disease [5]. Polypharmacy, according to the WHO, is a safe and effective treatment that includes at least five medications and is based on evidence. Unfortunately, combination medicines are frequently employed without scientific backing [6]. The benefit of polypharmacy versus monotherapy has been demonstrated in a number of well-known disease types, including cardiovascular disease [7], diabetes [8], as well as chronic pain [9,10].

The main aim of the review was to collect and include publications relating to clinical trials on the efficacy, tolerability, and rationale of beta-blockers and ACE-I combination use. Some data was based on the latest 2018 ESC/ESH Clinical Practice Guidelines for the Management of Arterial Hypertension and 2019 Principles of managing arterial hypertension of The Polish Society of Hypertension.

## 2. Data Sources and Study Selection

The search for publications on single-pill combinations to improve hypertension treatment was based on selecting those scientific reports that included assessing their efficacy and safety.

The databases of scientific literature—bibliographic were searched:

- The Medline;
- PubMed^®^;
- The Science Direct;
- The Cochrane Library (a database with systematic reviews).

Moreover, the following were checked:

- CENTRAL clinical trials registry;
- Drugs.com—an international drug information database;
- DrugBank—an international drug information database. The database is used by scientists and healthcare professionals around the world.

In the first stage, publications were automatically searched for using keywords and associated logical phrases, then manually selected for inclusion in the basic set.

Search keywords were the following: fixed-dose combination; single-pill combination; polytherapy of hypertension; hypertension treatment; hypertension; combination antihypertensive therapy; antihypertensive drugs; β-adrenergic receptors blockers; beta-blocker; angiotensin-converting enzyme inhibitors; ACE-I; beta-blockers and ACE-I combination; rationale of combining beta-blockers with ACE-I; and antihypertensive product overview.

The following items were removed from the basic set in stage 2:

- Publications unrelated to beta-blocker and ACE-I combinations (publications on monotherapy were excluded at the beginning);
- Publications on antihypertensive and other drug combinations that are not antihypertensive;
- Publications that describe circumstances that constitute indications for using combinations of the specified medications in a way that is overly vague and imprecise;
- Publications that were published prior to 1980;
- Publication of studies that were not randomized, placebo-controlled, or blinded (not meeting the GCP requirements). Selected publications were qualified in the third step based on the selection criteria applied. The papers included in the review were chosen based on whether the experiment was randomized, placebo-controlled, double-blind, or single-blind. They were carried out in accordance with good clinical practice (GCP) guidelines and designed in accordance with the Helsinki Declaration. In human research, they met medical, bioethical, and scientific requirements.

## 3. Hypertension Treatment

The primary goal of treating a patient with hypertension is to reduce mortality and the global risk of cardiovascular and renal complications. Lifestyle adjustments and pharmacological treatment are two well-established ways for lowering blood pressure. Although lifestyle changes can help lower blood pressure and, in some cases, cardiovascular risk, most people with hypertension will also need medication. Lifestyle changes refer to bodyweight reduction, a proper diet without saturated fat, increased consumption of vegetables and fruits, reduced consumption of alcohol and salt, no smoking, and increased regular physical activity. All guidelines agree that patients with grade 2 (160 > SBP > 179 mmHg and/or 100 > DBP > 109 mmHg) or grade 3 (SBP ≥ 180 mmHg and/or DBP ≥ 110 mmHg) hypertension, pharmacological treatment should be initiated immediately, together with lifestyle changes. For grade 1 hypertension (140 > SBP > 159 mmHg and/or 90 > DBP > 99 mmHg), non-pharmacological treatment should be instituted first, and in case of high cardiovascular risk, BP-lowering drugs should be added [1].

The ESC/ESH guidelines indicate that, in the light of many meta-analyses, the benefits of using essential antihypertensive drugs are comparable in reducing mortality and the total risk of cardiovascular complications, which results from the similar potency of their antihypertensive effect. A review of the scientific literature shows that despite many attempts in the 21st century, no new group of antihypertensive drugs that could improve the effectiveness of BP control, while reducing cardiovascular risk has been successfully introduced. Drugs from five groups remain in use and are recommended: thiazide/thiazide-like diuretics, β-adrenergic receptors blockers (commonly known as beta-blockers), calcium channel blockers, angiotensin-converting enzyme inhibitors (ACE-I), and drugs blocking the angiotensin II receptor (ARB) [1]. Nevertheless, two of the most commonly used antihypertension drugs are β-blocker and ACE-I. The above data is consistent with other recommendations [11,12].

### 3.1. β-Adrenergic Receptors Blockers (Beta-Blocker)

Beta-blockers are antagonists of β-adrenergic receptors (ARs), which play an important role in the regulation of physiological processes such as blood pressure, heart rate, and airway strength or reactivity, as well as metabolic and central nervous system functions [13]. The mechanism of the hypotensive action of beta-blockers is complex and may be the result of a reduction in the frequency of heart contractions and a decrease in cardiac output capacity, inhibition of renin secretion, increased baroreceptor reactivity, and blocking of β_2_ receptors in the presynaptic part of the sympathetic nervous system [14]. Tachycardia, hypertension, myocardial infarction, congestive heart failure, cardiac arrhythmias, coronary artery disease, hyperthyroidism, essential tremor, aortic dissection, portal hypertension, glaucoma, migraine prophylaxis, and other illnesses have all been approved by the FDA for treatment by β-blockers. [15].

Beta-blockers used in the hypertension treatment differ in their affinity for β_1_- and β_2_-adrenergic receptors. Some drugs selectively block β_1_-adrenergic receptors (cardioselective) and non-selectively block both β_1_ and β_2_ receptors [16] and are shown in Table 1.

The advantage of drugs that selectively block β_1_ receptors is a lower risk of bronchospasm and peripheral vasospasm (increased vascular resistance). Side effects are also a contraindication to the use of β-blockers, and they are decreased heart rate (bradycardia), disturbances in conduction in the heart’s conduction system (atrioventricular block), systolic bronchial conditions, peripheral vasoconstriction, diabetes, and hyperlipidemia [17].

Beta-blockers currently have uses other than cardiovascular medicine, such as migraine prophylaxis [18], the treatment of benign essential tremor [19], for patients with pheochromocytoma and thyrotoxicosis [20], and as topical ophthalmic formulations for lowering intraocular pressure in open angle glaucoma patients [21].

### 3.2. Angiotensin-Converting Enzyme Inhibitors (ACE-I)

Angiotensin-converting enzyme inhibitors are now widely used in the treatment of essential hypertension. The hypotensive effect of converting enzyme inhibitors results from the inhibition of angiotensin II production and the simultaneous inhibition of the degradation of endogenous vasodilating peptides (bradykinin and calidin), resulting in a significant BP reduction [22]. The beneficial effect on hemodynamics is expressed in reducing peripheral resistance with little or no effect on cardiac output [23]. The advantages of these drugs also include metabolic neutrality, potential antiatherosclerotic effects, and a beneficial effect on carbohydrate metabolism. Meta-analyses suggest an additional non-hypotensive effect of ACE-I in preventing cardiac complications, which may be related to the bradykinin effect of this group of drugs [24].

Depending on the mechanism of action, ACE-I can be divided into drugs that directly inhibit the activity of the angiotensin-converting enzyme (captopril, lisinopril) and prodrugs that are transformed in the liver, mainly by hydrolysis, into active ACE blocking metabolites (enalapril, trandolapril, quinapril, benazepril, perindopril, cilazapril, fosinopril, ramipril, imidapril, moexipril, and zofenopril) (Table 2).

The most common side effects include dry cough, occurring in 5–30% of patients, probably due to the inhibition of bradykinin degradation [25]. ACE-I can also induce hyperkalemia due to decreased aldosterone secretion, which is especially dangerous in patients concomitantly taking potassium-sparing diuretics or supplementing with potassium salts.

### 3.3. Single-Pill Combination

Poor control of cardiovascular risk factors has multiple causes, including shortcomings in the healthcare system in the area of cardiovascular prevention, physicians’ failure to use proven treatment strategies, therapeutic inertia (i.e., a lack of appropriate treatment changes when risk factor control is not achieved), and low adherence to prescribed lifestyle changes or drugs [26]. Therefore, it is extremely important to search for new pharmaceutical forms in the form of complex drugs. Literature data showed a connection between pill burden and medication adherence [27,28,29]. The better therapeutic response may also be a result of higher patient compliance [30].

The polypill approach is not exempted by potential inconveniences such as chemical incompatibility, physical instability, and pharmacokinetic properties of each component vs. the others [26]. The difference in drug solubility, but also a large dose difference, can reduce the availability of some drugs as well as, in part, their pharmacokinetic and pharmacodynamic characteristic [26].

Therefore, taking into account the possibility of interactions, it becomes extremely important to conduct a number of tests, such as stability tests, bioavailability tests, assessment of the short-term effects of the drugs on blood pressure, LDL cholesterol and platelet aggregation, assessment of safety and short-term symptomatic side effects, study of the interactions and effects of a combination of drugs on physiological mechanisms, and studies on adherence to treatment [31],which slows down the development of this modern pharmaceutical form.

### 3.4. Combination Antihypertensive Therapy

Significant and noticeable progress in the pharmacotherapy of arterial hypertension associated with increased blood pressure control in patients over the last dozen years is the more frequent use of antihypertensive drugs in the single-pill combination (SPC) form [32]. Observations from large clinical trials indicate that in approximately 60% of patients with hypertension, reasonable BP control can be achieved with two antihypertensive drugs at increasing doses. Another 20% achieve target BP with three antihypertensive drugs that good and long-term use follow the treatment recommendations.

**Figure 1:**
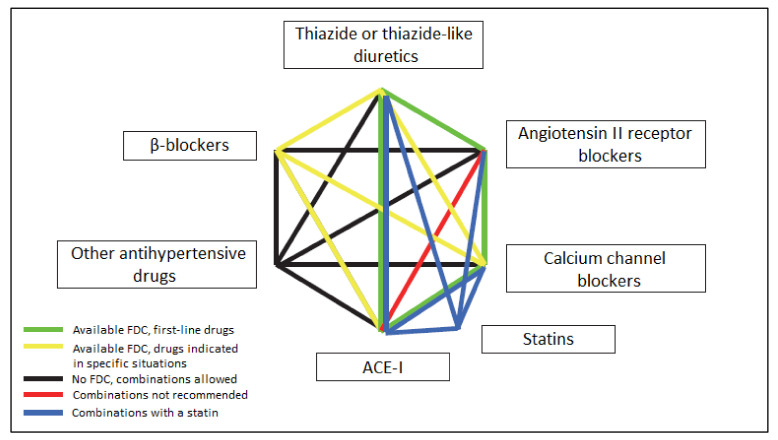
Combinations of antihypertensive drugs in dual-drug combination therapy, taking into account their usefulness and SPC availability [33].

Currently, there are eight types of two-drug SPCs. The basic two-drug combinations used to initiate the treatment of patients with uncomplicated hypertension, organ damage, metabolic disorders, or after a stroke include the following (Figure 1) [33]:

- ACE-I + calcium channel blockers;
- ACE-I + thiazide or thiazide-like diuretic;
- ARB + thiazide diuretic;
- ARB + calcium antagonist.

They are well-tolerated SPC drugs, effective in hypotension, reducing cardiovascular risk, and available worldwide in the form of combination preparations in a wide range of doses.

Moreover, two-drug combinations used in special situations of hypertension or in the case of multi-drug therapy, available worldwide in the SPC form, are:

- calcium channel blockers + beta-blocker;
- thiazide-like diuretic + calcium channel blockers;
- **beta-blocker + ACE-I**;
- thiazide diuretic + vasodilator beta-blocker.

The treatment strategy is based on the 2018 ESC/ESH and 2019 PSH guidelines, and it concentrates on five primary pharmacological classes: ACE inhibitors, ARBs, CCBs, thiazide or thiazide-like diuretics, and beta-blockers (Figure 1). The 2018 ESC/ESH guidelines show essential scientific and clinical evidence of combining two drugs in one pill, which gave rise to the idea of introducing single-pill combination (SPC) drugs [1], and the algorithm recommends initial therapy for most patients with that two drug-combination, ideally as an SPC. According to US Food and Drug Administration (FDA), SPC, previously called fixed-dose combination (FDC), is a term used for “a drug product in which two or more separate drug components (active pharmaceutical ingredients) are combined in a single dosage form” [34]. An SPC is considered rational if it is possible to provide scientific studies showing an increase in therapeutic efficacy, a reduction in adverse drug reactions, a reduction in individual doses and exposure, a reduction in the total therapy cost, a reduction in resistance or tolerance, or/and an improvement in patient adherence [35]. Besides the apparent increase in therapeutic efficacy, one of the more essential features of why SPCs were introduced into therapy was improving patients’ adherence by simplifying treatment regimens [36,37]. Not every combination of two drugs can be introduced as an SPC for therapeutic use. Before drugs can be combined into SPCs, several conditions must be met, including that drugs must act through different mechanisms of action, that the pharmacokinetic profiles of drugs must be similar, that drugs should not cause additive toxicity, and that the benefits of SPC mentioned above should be documented when using single drugs [35]. Still, SPC is more expensive than its constituents, even in the generic form [38]. Nevertheless, the better control and reduction of blood pressure and reduction of side effects reduces the overall cost of treatment by reducing the number of emergency room visits and hospitalizations compared to patients using single-pill combinations [39].

In 1980–2012, the FDA approved 117 non-new molecular entities SPC (non-NME-SPC) drugs, with the increased number over time with 12 approvals in the 1980s, 25 in the 1990s, 59 in the 2000s, and 21 in 2010s. The ATC classes with the most significant number of non-NME-SPC approved by the FDA were cardiovascular diseases (41), alimentary tract and metabolism (26), respiratory system (10), and antiinfectives (10) [40].

Polypharmacy has been shown to be more effective than monotherapy in the treatment of a variety of diseases, including arterial hypertension. For example, approximately 90% of hypertension patients in the Anglo-Scandinavian Cardiac Outcome Trial (ASCOT) required at least two antihypertensive medicines to attain blood pressure targets [8]. The additional antihypertensive benefit of combining medications from two distinct classes is almost five times stronger than doubling the dose of a single agent, according to a meta-analysis of 42 studies involving nearly 11,000 hypertensive patients [41].

### 3.5. Rationale of Combining Beta-Blockers with ACE-I

The most recent combination of a beta-blocker + ACE inhibitor in combination therapy (the only SPC available from 2015 in Europe is bisoprolol + perindopril) is recommended in the antihypertensive therapy of patients with hypertension and cardiac complications (coronary artery disease, also known as ischemic heart disease and heart failure). Such a combination is in line with 2015 PSH Guidelines and justified because, in these patients, regardless of myocardial infarction history, antihypertensive therapy should be based on such a combination [32]. Moreover, beta-blockers with ACE-I have become the mainstay of chronic heart failure treatment because both classes of drugs showed a morbidity and mortality reduction [42].

The principal indications for the usage of both categories of medicines support this. Beta-blockers are suggested as first-line therapy for angina and ischemia alleviation because they are effective at controlling symptoms in stable coronary artery disease (CAD), reducing exercise-induced angina, increasing exercise capacity, and decreasing ischemic episodes (both symptomatic and asymptomatic) [43]. As a result, ACE-I is indicated for the prevention of cardio-vascular risk factors such as hypertension, left ventricular failure, diabetes, and chronic kidney disease [43]. Therefore, a thorough neuroendocrine blockade is achieved by combining a beta-blocker and an ACE inhibitor. The beta-blocker component reduces cardiac output by blocking cardiovascular beta-adrenergic blockade, which is triggered by the SNS. The ACE inhibitor component stimulates vasodilation and lowers vascular resistance by acting on the renin-angiotensin system [44]. Further, an analysis of the EUROPA (European trial on Reduction Of cardiac events with Perindopril in stable coronary Artery disease) study showed that adding perindopril to a beta-blocker in patients with stable CAD was safe and reduced cardiovascular disease and mortality, i.e., had a lower risk of myocardial infarction and cardiovascular death compared to standard stable CAD therapy, including a single beta-blocker. This is a good reminder of the importance of providing stable CAD patients with pharmacotherapy that understands the blood vessel biology of atherosclerosis and respects the interaction of drugs [45]. What is essential is that bisoprolol with perindopril in SPC form is the only one approved concurrently for the three most considerable population therapeutic indications: hypertension, coronary artery disease, and heart failure.

Additionally, based on the Pol-Fokus study, it was noticed that the combination of a beta-blocker with an ACE-I is one of the most frequent combinations used by Polish doctors in the therapy combining two drugs [46]. Furthermore, this combination may be beneficial in younger patients with uncomplicated hyperkinetic hypertension (bisoprolol component) and signs and symptoms of organ damage, such as left ventricular hypertrophy (perindopril component) [32].

Although previous studies suggested that adding an angiotensin-converting enzyme inhibitor to a beta-blocker could cause an adverse reaction, a recent study and newly analyzed mechanistic data suggest that this combination provides modest additional angiotensin inhibition, which can help slow disease progression [47].

## 4. Combinations of Antihypertensive Drugs with Other Classes of Drugs

The high prevalence of arterial hypertension is associated with the frequent use of other classes due to comorbidities. In clinical practice, the most frequently prescribed drugs in treating hypertension treat hyperlipidemia, blood coagulation disorders, and Diabetes Mellitus type 2 (DM2) [48,49,50]. We often observe significant interactions or the intensification of the action of a given drug with intensive polytherapy due to the occurrence of action based on various mechanisms of action, often overlapping. The problem is fundamental in treating the elderly, who usually use polytherapy for many disease entities; usually, each individual is treated by a different specialist physician without regard to the entire therapy used by the patient. However, some drugs are considered the safe choice and used together because of the lack of significant interactions and sometimes the benefit of improving clinical response. Therefore, the development of single-pill combinations should be intensively consulted with clinicians experienced in the use of polytherapy.

### 4.1. Combination Treatment of Hypertension and Hyperlipidemia

The most significant observed problem in the treatment of hyperlipidemia is obtaining proper adherence and compliance on the part of the patient [51]. When treating hypertension or diabetes, the patient quickly begins to feel the adverse effects of discontinuing the prescribed therapy; however, the patient does not observe short-term side effects of discontinuing or suspending his therapy in hyperlipidemia [52]. The effect is visible only during routine checkups indicating significant disturbances in lipid metabolism. Due to the difficulty of obtaining good cooperation on the part of the patient, it seems crucial to use the fix-dosed formulation to obtain a better treatment response [53].

An important issue in selecting therapy in the presence of secondary diseases is the correct selection of drug groups due to the possibility of potential interactions. We do not observe any significant interactions between beta-blockers and ACE-I with statins, which are the most commonly used drugs in treating hyperlipidemia. In addition, research studies show the benefits of combining these groups of drugs [54,55,56]. Moreover, statins are considered the first treatment group in hyperlipidemia due to their excellent clinical response and a small number of side effects resulting from their use [57,58]. It is worth noting that there is a significant increase in cardiovascular diseases in the presence of hyperlipidemia.

For this reason, both diseases often coexist, which may lead to the willingness to develop single-pill combinations aimed at treating these disease entities [59,60]. The newly developed formulations show a relatively high degree of safety due to the extensive clinical experience in which these substances are used in the form of two tablets administered separately. The approved single-pill combinations are shown in Table 3.

### 4.2. Combination Treatment of Hypertension and Blood Coagulation Problems

Hypertension often goes hand in hand with the need to use anti-clotting medications to reduce the potential risk of lower limb venous thrombosis, strokes, and heart attacks. The gold standard in preventing myocardial infarction is administering low-dose acetylsalicylic acid (75–150 mg) thanks to the antithrombotic effect and the beneficial effects of the redox system [61]. An essential aspect of taking low doses of acetylsalicylic acid daily is the lack of side effects in deteriorating control of hypertension therapy [62]. Therefore, it seems crucial to develop new formulations based on the single-pill treatment regimen, taking into account the most commonly used drugs in treating hypertension with anticoagulant drugs, which bring significant benefits to most patients.

Patients with hypertension have a significantly increased risk of thromboembolic events. For this reason, we observe a significant percentage of combining classic antihypertensive therapy with drugs that reduce blood clotting, such as rivaroxaban, apixaban, or clopidogrel [63]. In addition, these drugs of choice are added to therapy to prevent subsequent episodes. The clinical trials showed significant benefits in reducing the risk of thrombotic events with a relatively low percentage of side effects when combining the therapy of hypertension with anticoagulant drugs [64,65,66]. However, current guidelines for the treatment of hypertension recommend the individual initiation of anticoagulants based on patient results and the assessment by the attending physician of the risk of thrombotic events [67].

### 4.3. Combination Treatment of Hypertension and Diabetes Mellitus Type II

It is estimated that approximately 50% of DM2 patients also have high blood pressure. It is estimated that diabetes doubles the risk of cardiovascular disease [68,69]. In developed countries, approximately 10% of cardiovascular events are due to diabetes [70]. This is a severe economic and social burden, especially considering the epidemiological forecasts indicating a significant increase in the prevalence of DM2 to 592 million by 2035 worldwide [71,72]. An important aspect to note is that hypertension is epidemiologically recognized as the cause of uncontrolled diabetes, and the conducted studies have shown a relationship between diabetes induction by hypertension [73,74,75].

The complexity of both disease entities, stabilization of pressure and carbohydrates metabolism, force the administration of at least a couple of medications to obtain an appropriate clinical response. Due to the necessity of administrating a few medications, pharmacotherapy becomes a heavy burden for the patient and the attending physician because of low adherence and patient compliance. ACE-I and diuretics are the drugs of choice for treating hypertension in diabetics [76,77,78]. Due to the necessity to take many medications, it seems necessary and justified to develop new single-pill formulations allowing for the limitation of the number of tablets required for a patient to take [79,80,81]. A significant problem in developing single-pill combinations is the significant individualization of therapy for each patient.

## 5. Single-Pill Combinations Drugs—Product Overview

Apart from scientific evidence regarding the possibility of using beta-blocker and ACE-I analogs combinations, there are not too many already registered products in the form of SPC. There is just one combination that is available in Europe; none of them are approved by FDA. However, there are many combinations of both groups with other antihypertensive drugs. Examples of SPCs approved by FDA and DCG are collected in Table 4.

Proving the validity of the combination of beta-blocker and ACE-I in hypertension treatment, it becomes necessary to develop this combination by pharmaceutical companies further to introduce a broader range of medicinal products in the form of SPC.

## 6. Pharmacoeconomics

Hypertension is associated with serious consequences and adds to substantial societal expenses. Total costs—the sum of direct and indirect costs, direct costs associated with therapy (drugs, medical equipment, diagnostics, hospitalization, consultations, etc.); indirect costs, i.e., presenteeism, absenteeism, and premature deaths; additional hospital costs, i.e., costs associated with the purchase and management of medications; pharmaceutical care costs; and out-of-pocket expenses, i.e., costs incurred directly by the patient [83]. Despite the availability of many effective drugs, still, approximately 30% of adult patients’ conditions worldwide are poorly controlled, which in turn increases the total healthcare costs [1]. The increasing role of FDC therapy in hypertension is a crucial strategy to reduce treatment failure [84]. Fixed-dose combination drugs as a SPC (single pills combination) in hypertension have many positive pharmacoeconomics aspects. This effect is largely due to improved adherence and persistence, a characteristic of SPC. Adherence and persistence is higher in patients taking antihypertensives as SPC vs. a free-dose combination. Many studies showed that SPCs improved adherence to antihypertensive medications compared with FEC in adults aged > 18 years [37]. SPC improved clinical outcome parameters and led to better clinical outcomes under conditions, which directly affects the economic benefits [85,86,87]. Persistence in the SPCs groups was twice as likely as in the FEC groups [88]. The positive effect of SPCs is likely higher in the real-world setting. This pharmacoeconomics effects of adherence and significantly persistence of SPCs use also include the reduction of hospitalizations in patients with hypertension [89]. Using SPC rather FDC may represent potentially low-cost intervention. It is an essential conclusion regarding public health—medical decisions, medicines’ affordability, and drug reimbursement could reduce the global burden of death and disability related to hypertension [90].

A population-based retrospective cohort study reports that higher costs of SPC drugs are compensated by reducing overall medical costs and optimizing spending in the healthcare system. However, the availability of generic medications should be considered. It is critical to use similarly safe and effective lower-cost generic medications to decrease wasteful hypertension spending in health-care systems [38]. Less treatment complexity, accelerated time to control, improved patient adherence, fewer side effects, and reduced therapy inertia are all examples of cost reductions in hypertension treatment [1]. Significant benefits for health care systems have been identified, taking into account public spending for SPCs and FDC, e.g., [91,92,93,94,95,96,97,98,99]:

- valsartan/amlodipine;
- valsartan/hydrochlorothiazide;
- indapamide/amlodipine;
- olmesartan/amlodipine;
- amlodipine/valsartan/hydrochlorothiazide;
- atorvastatin/perindopril/amlodipine;
- olmesartan/amlodipine/hydrochlorothiazide.

Using the Markov model to analyze quality-adjusted life years (QALYs), a high cost-effectiveness SPCs compared to free-drug combination therapy was proven. Potential cost-saving revisions in treatments should consider adherence effects, resulting in an economic evaluation of the pharmacotherapy [100]. However, some studies show that, despite the clinical effects, not every drug combination necessarily has the same cost-effectiveness [99]. Improving hypertension treatment strategies, including the use of SPCs, is necessary nowadays. Increasing the effectiveness of hypertension treatment in the US would prevent 389,000 to 478,000 CVD events per year.

Interestingly, treating all stage 1 hypertension would cost more than treating only >5% CVD risk [101]. Implementation of a cost-effective hypertension control program should include SPC combined with additional measures to build patient competence and attitudes, such as motivational support with patients [102]. SPC therapy as a first-line therapy should be implemented as a hypotensive treatment strategy in order to effectively spend public funds on healthcare [1,103]. There should be strong national guidelines for better hypertension control and pharmacoeconomics effects. The implementation of SPCs is also recommended for low- and middle-income countries, where particular attention should be given to the pooled procurement of low-cost treatments. Global Hypertension Practice Guidelines recommended by the International Society of Hypertension suggest that hypertension treatment should be affordable and/or cost-effective relative to other agents. So, all countries should have single-pill combinations in their own essential medicines lists to ensure cost-effective and patient-centered health policies [104]. When introducing SPC to the reimbursement system, however, the pharmacoeconomic effectiveness should be assessed each time. Systematic review shows that SPC improved BP outcomes compared with FECs [87]. However, there is no doubt that the use of SPC in hypertension plays a significant role in reducing global cardiovascular morbidity and mortality.

## 7. Conclusions

Even The 2018 European Society of Cardiology/European Society of Hypertension Guidelines advocate single-pill combo medicines for hypertension management. According to research, compared to starting free-dose combination medication, this therapy improves adherence, blood pressure control, and/or cardiovascular outcomes. Many new drug combinations have been approved, allowing the development of new single-pill formulations. The observed trend indicates a significant increase in the combined preparations available for pharmacotherapy in the coming years.

Notably, the use of single-pill drugs seems to be optimal in the treatment of hypertension and in concomitant diseases such as hyperlipidemia, blood coagulation problems, and diabetes. In an extensive range of patients, these diseases are also common as caused by the underlying disease. For this reason, it is necessary to significantly concentrate the pharmaceutical industry and scientists in the development of complex formulations allowing the treatment of many disease entities.

## Figures and Tables

**Table 1:** Chemical structures of non-selective and selective β-blockers.

Non-Selective β_1_/β_2_ Agents				
Bucindolol ^1^ 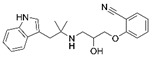	carteolol 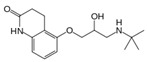	Carvedilol ^1,3^ 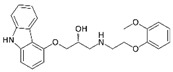	Labetalol ^1,3^ 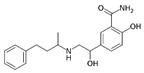	nadolol 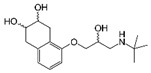
Oxeprenolol ^2^ 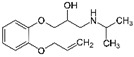	Penbutolol ^2^ 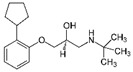	Pindolol ^2^ 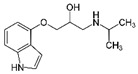	propranolol 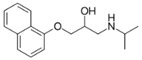	timolol 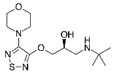
β_1_-selective agents				
acebutolol 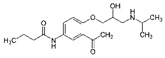	atenolol 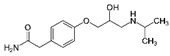	betaxolol 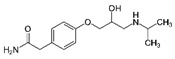	bisoprolol 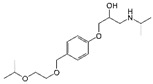	celiprolol 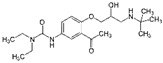
	esmolol 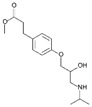	metoprolol 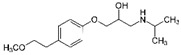	Nebivolol ^3^ 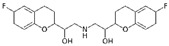	

^1^ has additional α_1_-blocking activity. ^2^ has intrinsic sympathomimetic activity. ^3^ has a vasoconstrictor effect.

**Table 2:** Chemical structures of ACE-I.

Drugs Directly Inhibit the Activity of the Angiotensin Converting Enzyme			
	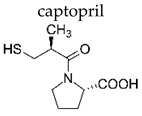	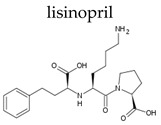	
Prodrugs and its active forms			
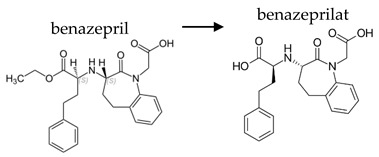		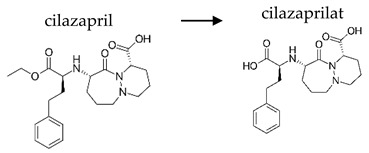	
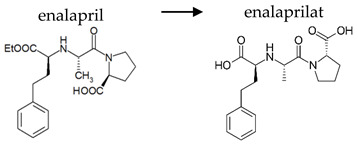		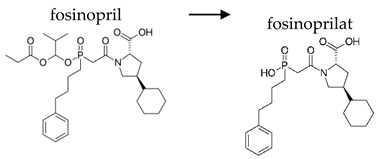	
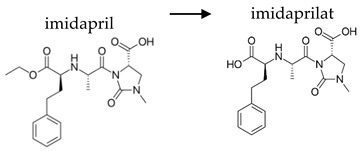		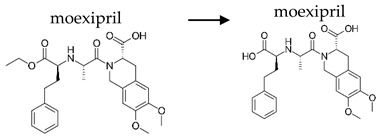	
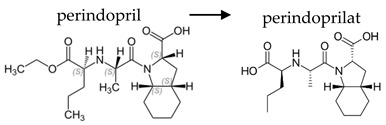		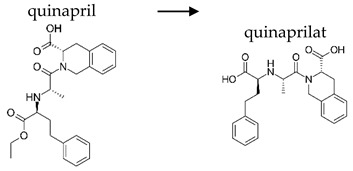	
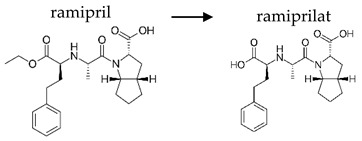		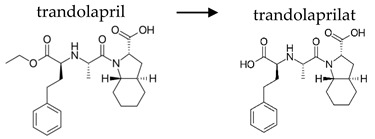	
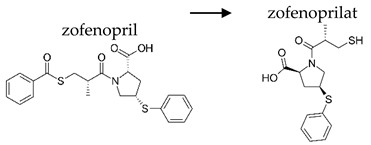			

**Table 3:** Clinical trials.

Name of Clinical Trial	Randomized Patients	Studied Drugs	Effect	Literature
EUROPA	12,218	Perindopril + beta-blocker	reduced cardiovascular disease and mortality	[32]
Pol-Fokus	12,375	ACE-I + beta-blocker	the most commonly prescribed drug combination	[33]

**Table 4:** Approved drugs with beta-blocker or ACE-I and beta-blocker with ACE-I [34,82].

Name of Drugs	Date of Approval (FDA)	Date of Approval (DCG)
SPC with beta-blockers		
Atenolol 50 mg + Nifedipine 20 mg	-	1990
Timolol maleate 25 mg + Hydrochlorothiazide 10 mg	1981 *	-
Metoprolol tartrate 50 mg + Hydrochlorothiazide 25 mg	1984	1982
Propranolol hydrochloride 40/80 mg + Hydrochlorothiazide 25 mg	1987	1982
Propranolol hydrochloride 80 mg + Bendrafluazide 2.5 mg	-	1982
Nadolol 40/80 mg + Bendroflumethiazide 5 mg	1983	-
Acebutolol hydrochloride 200 mg + Hydrochlorothiazide 12.5 mg	-	1986
Labetalol hydrochloride 100/200/300/400 mg + Hydrochlorothiazide 25 mg	1987 *	-
Metoprolol tartrate 100/200 mg + Chlorthalidone 25 mg	1987 *	-
Atenolol 25/50/100 mg + Chlorthalidone 6.25/12.5/25 mg	1990	2008
Betaxolol hydrochloride 5/10 mg + Chlorthalidone 12.5 mg	1992 *	-
Pindolol 5/10 mg + Hydrochlorothiazide 25 mg	1995 *	-
Atenolol 30 mg + Nitrendipine 10/20 mg	-	1995
Atenolol 25/50 mg + Amlodipine besylate 2.5/5 mg	-	1996
Bisoprolol fumarate 2.5/5/10 mg + Hydrochlorothiazide 6.25 mg	2000	1999
Atenolol 50 mg + Lercanidipine 10 mg	-	2003
Nabivolol hydrochloride 5 mg + Hydrochlorothiazide 12.5 mg	-	2004
Atenolol 50 mg + Losartan 50 mg	-	2005
Atenolol 25/50 mg + Hydrochlorothiazide 12.5/25 mg	-	2005
Bisoprolol 2.5/5 mg + Amlodipine 5 mg	-	2005
Metoprolol succinate 25/50/100 mg + Hydrochlorothiazide 12.5 mg	2006	2005
Nebivolol hydrochloride 5 mg + Valsartan 80 mg	2016 *	2006
Nevibolol hydrochloride 5 mg + Amlodipine besylate 5/10 mg	-	2006
Atenolol 25/50 mg + Indapamide 1.5 mg	-	2007
Nebivolol 2.5/5 mg + Indapamide 1.5 mg	-	2010
Metoprolol succinate 25/50 mg + Telmisartan 20/40 mg	-	2010
Carvedilol 6.25/12.5/25 mg + Ivabradine hydrochloride 5/7.5 mg	-	2018
**SPC with ACE-I**		
Enalapril maleate 5/10 mg + Hydrochlorothiazide 12.5/25 mg	1986	1992
Captopril 25/50 mg + Hydrochlorothiazide 15/25 mg	-	1989
Benazepril hydrochloride 5/10/20 mg + Hydrochlorothiazide 6.25/12.5/25 mg	1992	1998
Fosinopril sodium 10/20 mg + Hydrochlorothiazide 12.5 mg	1994	-
Benazepril hydrochloride 10/20/40 mg + Amlodipine besylate 2.5/5/10 mg	1995	2002
Enalapril Maleate 2.5/5 mg + Amlodipine 2.5/5 mg/2.5	-	1995
Lisinopril 5/10/20 mg + Hydrochlorothiazide 12.5/25 mg	-	1995
Enalapril maleate 5 mg + Felodipine 2.5/5 mg	1996	-
Trandolapril 1/2/4 mg + Verapamil hydrochloride 180/240 mg	1996	2006
Ramipril 2.5/5 mg + Hydrochlorothiazide 12.5/25 mg	-	1996
Moexipril hydrochloride 7.5/15 mg + Hydrochlorothiazide 12.5/25 mg	1997	-
Lisinoril 5 mg + Amlodipine besylate 2.5/5 mg	-	1997
Perindopril 2/4 mg + Indepamide 0.625/1.25 mg	-	1998
Quinapril hydrochloride 10/20 mg + Hydrochlorothiazide 12.5/25 mg	1999	2003
Ramipril 2.5/5/10 mg + Amlodipine 2.5/5 mg	-	2003
Ramipril 2.5/ 5 mg + Candesartan cilexatil 8 mg	-	2003
Ramipril 2.5/5 mg + Losartan Potassium 50 mg	-	2003
Ramipril 2.5/5 mg + Telmisartan 40 mg	-	2005
Enalapril 2.5/5 mg + Hydrochlorothiazide 12.5 mg	-	2005
Perindopril arginine 3.5/7/14 mg + Amlodipine besylate 2.5/5/10 mg	2015	2007
Ramipril 2.5/5 mg + Atorvastatin Calcium 10/20 mg	-	2007
Ramipril 5 mg + Olmesartan medoximil 20/40 mg	-	2007
Ramipril 2.5/5 mg + Metolazone 2.5 mg	-	2008
Ramipril 2.5 mg + Indapamide 1.5 mg	-	2009
**SPC with beta-blockers and ACE-I**		
Metoprolol succinate 25/50 mg + Ramipril 2.5/5 mg	-	2007
Metoprolol 25/50 mg + Ramipril 2.5/5/10 mg + Atorvastatin 10/20 mg	-	2009
Atenolol 50 mg + Ramipril 5 mg + Simvastatin 20 mg + Hydrochlorothiazide 12.5 mg	-	2009
Atenolol 25/50 mg + Lisinopril 5/10 mg + Simvastatin 10/20 mg	-	2010
Bisoprolol 5/10 mg + Perindopril 5/10 mg	Available in EU from 2015	
**SPC with beta-blockers and/or ACE-I and statins**		
Metoprolol 25/50 mg + Atorvastatin 10 mg	-	2010
Metoprolol Succinate 25/50 mg + Atorvastatin 10 mg	-	2008
Metoprolol 50/50/25 mg + Atorvastatin 20/10/10 mg + Ramipril 10/5/2.5 mg	-	2009
Simvastatin 20 mg + Ramipril 5 mg + Atenolol 50 mg + HCTZ 12.5 mg + Aspirin 100 mg	-	2009
Metoprolol Succinate 25/50 mg + Atorvastatin 10 mg	-	2008
Atorvastatin 10 mg + Ramipril 5 mg + Aspirin 75/150 mg + Metoprolol 25 mg	-	2010
Aspirin 75 mg + Simvastatin 10/20 mg + Lisinopril 5/10 mg + Atenolol 25/50 mg	-	2010
Atorvastatin Calcium 10 mg + Ramipril 2.5/5 mg	-	2007
Atorvastatin 20 mg + Ramipril 2.5/5 mg	-	2010
**SPC with beta-blockers and/or ACE-I and anticoagulant drugs**		
Simvastatin 20 mg + Ramipril 5 mg + Atenolol 50 mg + HCTZ 12.5 mg + Aspirin 100 mg	-	2009
Atorvastatin 10 mg + Ramipril 5 mg + Aspirin 75/150 mg + Metoprolol 25 mg	-	2010
Metoprolol tartarate 25/50 mg + Clopidogrel 75 mg	-	2010
**SPC with beta-blockers and/or ACE-I and antidiabetic drugs**		
No formulation has been accepted	-	-

* discontinued.

## Data Availability

Not applicable.
